# 重型再生障碍性贫血儿童接受异基因造血干细胞移植前铁过载对疗效影响的临床研究

**DOI:** 10.3760/cma.j.cn121090-20231214-00310

**Published:** 2024-06

**Authors:** 黎 晏, 昊 熊, 飞 龙, 智 陈, 卓 王, 李 杨, 芳 陶, 燕 陈, 娜 宋, 旻 吴

**Affiliations:** 1 华中科技大学同济医学院附属武汉儿童医院儿童血液疾病研究室，武汉 430016 Laboratory of Pediatric Hematology, Wuhan Children's Hospital Affiliated to Tongji Medical College, Huazhong University of Science and Technology, Wuhan 430016, China; 2 华中科技大学同济医学院附属武汉儿童医院儿童血液科，武汉 430016 Department of Hematology, Wuhan Children's Hospital Affiliated to Tongji Medical College, Huazhong University of Science and Technology, Wuhan 430016, China

## Abstract

探讨铁过载对治疗重型再生障碍性贫血（SAA）患儿异基因造血干细胞移植（allo-HSCT）疗效的影响。通过对武汉儿童医院血液科2018年1月至2022年8月间接受allo-HSCT的74例SAA患儿的临床资料进行回顾性分析，发现移植前铁过载组［血清铁蛋白（SF）>1 000 µg/L］患儿移植前病程更长、红细胞输注量更大、回输CD34^+^细胞数更多，且铁过载显著延迟了移植后调节性T细胞（Treg细胞）的重建，增加了移植后出血性膀胱炎和Ⅲ～Ⅳ度急性移植物抗宿主病的发生率。然而，铁过载对患儿的总生存率和无失败生存率并无显著影响。

## Abstract (en)

This study aimed to investigate the effect of iron overload on the transplant outcomes of pediatric patients with severe aplastic anemia（SAA）undergoing allogeneic hematopoietic stem cell transplantation（allo-HSCT）. A retrospective analysis was conducted on the clinical data of 74 children with SAA who received allo-HSCT at the Hematology Department of Wuhan Children's Hospital between January 2018 and August 2022. Children with iron overload（serum ferritin >1 000 µg/L）before transplantation had a longer disease course, received more red blood cell transfusions, and had a higher number of CD34^+^ cells infused. Moreover, iron overload significantly delayed the reconstitution of regulatory T cells after transplantation, increasing the incidence of hemorrhagic cystitis and grade Ⅲ–Ⅳ acute graft-versus-host disease after transplantation. However, iron overload did not significantly affect the overall survival and failure-free survival rates of the children.

异基因造血干细胞移植（allo-HSCT）是目前治疗儿童重型再生障碍性贫血（SAA）的主要手段[1]。接受移植的SAA患儿如长期输血治疗，罹患铁过载风险显著增加[2]。既往研究发现铁过载是急性髓系白血病（AML）和骨髓增生异常综合征（MDS）移植预后不良的危险因素，但目前SAA患儿移植前铁过载对预后影响的研究相对较少[2]–[4]。本研究回顾性分析武汉儿童医院血液科接受allo-HSCT治疗的SAA患儿临床资料，旨在探索铁过载对SAA患儿移植疗效的影响。

## 病例与方法

1. 病例资料：回顾性收集2018年1月1日至2022年8月31日在武汉儿童医院血液科接受allo-HSCT的74例SAA患儿临床资料。诊断及分型标准参照《再生障碍性贫血诊断与治疗中国指南（2022年版）》[5]。根据《铁过载诊断与治疗的中国专家共识》[6]，取受试者移植前血清标本，采用化学发光免疫分析法测定血清铁蛋白（SF）水平，结合既往输血史和C反应蛋白水平排除SF假性升高，以SF>1 000 µg/L定义为铁过载。本研究获得华中科技大学附属武汉儿童医院伦理委员会批准（批件号：2022R071-E01），所有参与研究患儿的监护人均书面签署相关检查治疗的知情同意。

2. 造血干细胞来源：所有患儿移植物均为来自外周血的造血干细胞。供者以皮下注射G-CSF 10 µg·kg^−1^·d^−1^的方式进行造血干细胞动员，连用5 d，期间监测血常规，第5 d接受G-CSF治疗2 h后进行采集。回输采集物的单个核细胞数计数控制在（6～10）×10^8^/kg，CD34^+^细胞计数>2×10^6^/kg。

3. 预处理方案：亲缘单倍体及无关供者移植采用氟达拉滨（Flu）+环磷酰胺（Cy）+兔抗人胸腺细胞免疫球蛋白（ATG）方案，其中Flu：50 mg·m^−2^·d^−1^×4 d；Cy：60 mg·kg^−1^·d^−1^×2 d；ATG总量为7.5 mg/kg，分3 d给药。同胞全相合患儿Flu与上述剂量相同，Cy为50 mg·kg^−1^·d^−1^×4 d，ATG总量为10 mg/kg，分3 d给药。

4. 移植物抗宿主病（GVHD）的预防：采用环孢素A（CsA）+霉酚酸酯（MMF）+短程甲氨蝶呤（MTX）方案：MTX 15 mg·m^−2^·d^−1^，+1 d；10 mg·m^−2^·d^−1^，+3、+6 d。CsA预处理开始1.5 mg·kg^−1^·d^−1^持续静脉滴注20 h，−1 d改为3 mg·kg^−1^·d^−1^持续静脉滴注20 h，植入稳定后改为口服，维持谷浓度150～250 µg/L；如患儿未发生GVHD，移植后9～12个月逐渐减量，至移植后13～15个月减停。

5. 预防感染与支持治疗：所有患儿均在百级层流病房接受预处理至造血功能完成重建。移植后口服复方磺胺甲唑预防肺孢子菌病；移植期间应用米卡芬净或卡泊芬净预防真菌感染，移植后改为口服泊沙康唑；预处理前使用更昔洛韦预防病毒感染，移植期间静脉输注阿昔洛韦预防病毒感染，造血重建后改用口服阿昔洛韦。对症给予碱化、水化、止吐、保肝、保护黏膜和预防感染。移植期间密切监测血常规，按照输血标准给予成分输血。

6. 淋巴细胞亚群检测：采用MultiTEST IMK Kit试剂盒（美国BD公司）检测患儿移植后1、2、3、6及12个月外周血淋巴细胞亚群绝对计数，包括CD3^+^ T细胞、CD4^+^ T细胞、CD8^+^ T细胞、NK细胞、CD19^+^ B细胞、调节性T细胞（Treg细胞）。

7. 相关定义及随访：中性粒细胞植入定义为连续3 d ANC≥0.5×10^9^/L；血小板植入定义为在未输注血小板的前提下，连续7 d PLT≥20×10^9^/L；植入失败包括原发性植入失败和继发性植入失败。急性GVHD（aGVHD）和慢性GVHD（cGVHD）的诊断及分级标准参照2020年欧洲血液和骨髓移植学会（EBMT）共识[7]。总生存（OS）定义为移植到死亡或末次随访的时间；无失败生存（FFS）定义为有治疗反应的生存时间，死亡、植入失败和复发被认为是治疗失败。所有患者随访至2023年9月30日。随访方式包括查阅病历、电话随访。

8. 统计学处理：采用SPSS 22.0进行统计分析，GraphPad prism 8.0绘图。非正态分布数据以*M*（*IQR*）表示，计数资料以例数（构成比）表示。计数资料组间比较采用*χ*^2^检验或Fisher精确概率法；非正态分布数据组间比较采用Mann-Whitney *U*检验。采用Kaplan-Meier法进行生存分析，组间比较采用Log-rank检验。双侧*P*<0.05为差异有统计学意义。

## 结果

1. 一般资料：74例患儿中男33例，女41例，接受移植时中位月龄为71（46，101）个月，其中铁过载组25例（33.8％），非铁过载组49例（66.2％）。铁过载组与非铁过载组SF的中位水平分别为1 424（1 245，1 657）µg/L和474（343，598）µg/L（*P*<0.001）。两组患儿在性别、年龄、疾病类型、移植前ECOG评分、移植前HSCT合并症指数（HCT-CI）、移植前血小板输注量、供受者性别、供受者血型相合、移植方式和回输单个核细胞数方面差异均无统计学意义（均*P*>0.05）。与非铁过载组相比，铁过载组患儿移植前病程更长、红细胞输注量更大、回输CD34^+^细胞数更多，差异均有统计学意义（均*P*<0.05）（表1）。

**表1:** 铁过载组与非铁过载组重型再生障碍性贫血患儿一般临床特征

临床特征	铁过载组（25例）	非铁过载组（49例）	*P*值
血清铁蛋白[µg/L，*M*（*IQR*）]	1424（1245, 1657）	474（343, 598）	<0.001
性别[例（%）]			0.159
男	14（56.0）	19（38.8）	
女	11（44.0）	30（61.2）	
月龄[月，*M*（*IQR*）]	82（46, 122）	67（43, 86）	0.142
疾病类型[例（%）]			0.279
SAA	18（72.0）	29（59.2）	
VSAA	7（28.0）	20（40.8）	
确诊至移植时间[月，*M*（*IQR*）]	6.0（3.0, 9.5）	3.0（2.0, 4.8）	<0.001
移植前ECOG评分[例（%）]			0.761
0~1分	23（92.0）	46（93.9）	
2~3分	2（8.0）	3（6.1）	
移植前HCT-CI评分[例（%）]			0.852
0分	22（88.0）	43（87.8）	
1~2分	2（8.0）	5（10.2）	
≥3分	1（4.0）	1（2.0）	
移植前红细胞输注量[U，*M*（*IQR*）]	8.0（6.5, 11.2）	6.5（4.0, 8.5）	0.028
移植前血小板输注量[U，*M*（*IQR*）]	8.0（4.7, 14.2）	6.5（3.5, 9.0）	0.066
预处理前血常规[*M*（*IQR*）]			
ANC（×10^9^/L）	0.24（0.05, 0.59）	0.28（0.12, 0.68）	0.501
HGB（g/L）	85（67, 91）	73（65, 83）	0.056
PLT（×10^9^/L）	22（8, 66）	28（12, 50）	0.824
预处理前骨髓增生程度[例（%）]			0.528
减低	20（80.0）	42（85.7）	
重度减低	5（20.0）	7（14.3）	
供受者性别[例（%）]			0.164
男供男	9（36.0）	12（24.5）	
男供女	10（40.0）	18（36.7）	
女供男	5（20.0）	7（14.3）	
女供女	1（4.0）	12（24.5）	
供受者血型相合[例（%）]			0.353
相合	8（32.0）	22（44.9）	
主要不合	6（24.0）	6（12.2）	
次要不合	7（28.0）	17（34.7）	
主次均不合	4（16.0）	4（8.2）	
移植方式[例（%）]			0.367
同胞全相合移植	2（8.0）	6（12.2）	
单倍体移植	9（36.0）	24（49.0）	
无关供者移植	14（56.0）	19（38.8）	
预处理ATG用量[例（%）]			0.665
10 mg/kg	14（56.0）	30（61.2）	
7.5 mg/kg	11（44.0）	19（38.8）	
回输MNC细胞数[10^8^/kg，*M*（*IQR*）]	8.00（6.70,9.36）	8.05（7.70,8.67）	0.716
回输CD34^+^ 细胞数[10^6^/kg，*M*（*IQR*）]	6.16（4.19,7.93）	4.70（3.53,7.06）	0.031

**注** SAA：重型再生障碍性贫血；VSAA：极重型再生障碍性贫血；ECOG：美国东部肿瘤协作组；HCT-CI：造血干细胞移植合并症指数，ATG：抗胸腺细胞免疫球蛋白；MNC：单个核细胞

2. 免疫重建：与非铁过载组相比，铁过载组患儿在移植后第1个月Treg细胞数量明显减少［3（0，6）个/µl对11（1，32）个/µl，*P*＝0.045］。两组CD3^+^ T、CD4^+^ T、CD8^+^ T、CD19^+^ B和NK细胞数量在移植后第1、2、3、6、12个月差异均无统计学意义（均*P*>0.05）（表2）。

**表2:** 铁过载与非铁过载组重型再生障碍性贫血患儿移植后不同时间各淋巴细胞亚群绝对计数的比较［个/µl，*M*（*IQR*）］

组别	移植后时间				
	1个月	2个月	3个月	6个月	12个月
铁过载组					
CD3^+^ T细胞	43（6, 368）	836（304, 1 734）	1012（442, 1 770）	1068（710, 1 441）	2150（1 638, 2 631）
CD4^+^ T细胞	25（1, 72）	138（66, 273）	117（74, 232）	181.5（134, 357）	768（455, 1 002）
CD8^+^ T细胞	37（2, 254）	605（236, 1 183）	605（236, 1 183）	702（436, 1 152）	1141（781, 1 431）
NK细胞	139（67, 416）	224（130, 362）	294（141, 1 021）	383（158, 564）	257（145, 459）
CD19^+^ B细胞	1（0, 3）	4（1, 95）	39（3, 155）	101（38, 322）	396（211, 572）
Treg细胞	3（0, 6）^a^	13（4, 23）	10（7, 21）	26（10, 38）	65（32, 89）
非铁过载组					
CD3^+^ T细胞	80（15, 680）	1417（416, 2 476）	1088（460, 2 209）	1239（555, 2 009）	2064（1 663, 2 862）
CD4^+^ T细胞	39.0（4, 112）	210（92, 339）	191（102, 298）	280（154, 424）	663（483, 776）
CD8^+^ T细胞	37（6, 360）	769（221, 1 814）	1012（456, 1 805）	916（386, 1 432）	1399（904, 2 051）
NK细胞	193（92, 193）	310（188, 590）	328（177, 638）	254（114, 593）	292（202, 477）
CD19^+^ B细胞	2（1, 5）	9（0, 124）	14（1, 124）	79（8, 200）	237（112, 384）
Treg细胞	11（1, 32）	10（3, 20）	10（5, 24）	18（8, 35）	40（30, 63）

**注** Treg细胞：调节性T细胞；a：与非铁过载组比较，*P*<0.05

3. 造血重建：铁过载组与非铁过载组中性粒细胞中位植入时间分别为13（12，15）d和13（12，14）d，血小板植入时间分别为14（12，21）d和14（12，18）d，差异均无统计学意义（*P*值分别为0.776、0.934）。两组28 d内中性粒细胞植入成功率均为100.0％，血小板植入成功率分别为95.8％和95.5％。铁过载组1例患儿发生植入失败，造血持续未恢复，于+146 d死于脓毒性休克。非铁过载组1例患儿发生植入失败，于+85 d死于血栓性微血管病（TMA）。

4. 感染和并发症：铁过载组移植后+29～+365 d内发生CMV血症15例（60.0％），EBV血症13例（52.0％），脓毒血症11例（44.0％），肺部感染9例（36.0％）；而非铁过载组中发生CMV血症20例（40.8％），EBV血症28例（57.1％），脓毒血症12例（24.5％），肺部感染23例（46.9％）。铁过载组移植后CMV血症发生率高于非铁过载组（60.0％对40.8％），但差异无统计学意义（*P*＝0.144）（表3）。铁过载组移植后发生出血性膀胱炎（HC）8例（32.0％），TMA 2例（8.0％），移植后淋巴增殖性疾病（PTLD）1例（4.0％）；非铁过载组发生HC 5例（10.2％），TMA 5例（10.2％），PTLD 3例（6.1％），肝静脉闭塞病（VOD）2例（4.1％）。与非铁过载组相比，铁过载组HC发生率显著升高（32.0％对10.2％，*P*＝0.027）（表3）。

**表3:** 铁过载组与非铁过载组重型再生障碍性贫血患儿移植后不良事件的比较［例（％）］

不良事件	铁过载组（25例）	非铁过载组（49例）	*P*值
植入失败	1（4.0）	1（2.0）	0.659
aGVHD	10（40.0）	19（38.8）	0.972
Ⅰ~Ⅱ度	4（16.0）	17（34.7）	0.562
Ⅲ~Ⅳ度	6（24.0）	2（4.1）	0.016
cGVHD	2（8.0）	5（10.2）	1.000
感染			0.343
肺部感染	9（36.0）	23（46.9）	
脓毒血症	11（44.0）	12（24.5）	
消化道感染	6（24.0）	11（22.4）	
上呼吸道感染	2（8.0）	8（16.3）	
CMV血症	15（60.0）	20（40.8）	0.144
EBV血症	13（52.0）	28（57.1）	0.674
移植后并发症			
HC	8（32.0）	5（10.2）	0.020
TMA	2（8.0）	5（10.2）	0.759
PTLD	1（4.0）	3（6.1）	0.703
VOD	0（0.0）	2（4.1）	0.306
死亡	3（12.0）	6（12.2）	1.000

**注** aGVHD：急性移植物抗宿主病；cGVHD：慢性移植物抗宿主病；CMV：巨细胞病毒；EBV：EB病毒；HC：出血性膀胱炎；TMA：血栓性微血管病；PTLD：移植后淋巴增殖性疾病；VOD：肝静脉闭塞病

5. GVHD：铁过载组中有10例（40.0％）患儿发生aGVHD，其中Ⅰ～Ⅱ度4例（16.0％），Ⅲ～Ⅳ度6例（24.0％）。非铁过载组有19例（38.8％）患儿发生aGVHD，其中Ⅰ～Ⅱ度17例（34.7％），Ⅲ～Ⅳ度2例（4.1％）。与非铁过载组相比，铁过载组Ⅲ～Ⅳ度aGVHD发生率显著增加（24.0％对4.1％，*P*＝0.016）（表3）。两组分别有2例（8.0％）和5例（10.2％）患儿发生cGVHD，差异无统计学意义（*P*＝1.000）（表3）。

6. 生存：中位随访17（12～26）个月，74例患儿中65例（87.8％）存活，9例（12.2％）死亡。铁过载组3例死亡，其中2例死于TMA，1例死于脓毒性休克；非铁过载组6例死亡，其中3例死于TMA，2例死于脓毒性休克，1例死于多脏器功能衰竭。两组2年OS率分别为（87.5±6.8）％和（88.6±4.8）％（*P*＝0.892），FFS率分别为（83.3±7.6）％和（88.6±4.8）％（*P*＝0.710），差异均无统计学意义。

## 讨论

近年来随着移植技术的飞速发展和移植相关支持治疗方法的不断改进，积极接受allo-HSCT治疗的SAA患儿逐渐增多。如何降低移植后并发症和移植相关死亡率是目前临床面临的重要挑战。

SAA患儿因长期依赖输血治疗，导致铁过载现象常见。铁过载的危害主要是诱导产生大量活性氧自由基，促进细胞坏死或凋亡，引起多器官功能损伤[3]–[4]。本研究发现，两组患儿allo-HSCT后粒细胞、血小板植入时间并无明显差异，但铁过载组Treg细胞重建显著延迟。Treg细胞是一类具有免疫负调节功能的T细胞亚群，在维持自身免疫平衡、诱导移植耐受中发挥重要作用[8]。Edinger等[9]在骨髓移植后小鼠模型中发现，CD4^+^CD25^+^Treg细胞能够抑制供者早期T淋巴细胞扩增，进而降低GVHD的发生率。与效应T细胞相比，CD4^+^Treg细胞的重建主要通过受者外周淋巴器官扩增实现，在allo-HSCT后6周内比例可恢复正常[10]。本研究中SAA患儿体内过度沉积的铁或可诱导外周淋巴器官功能损伤，影响患儿Treg细胞重建，导致感染、复发、严重GVHD等并发症。进一步研究铁过载与Treg细胞重建规律，可能为降低移植后并发症提供新思路。

铁过载是移植后aGVHD发生率增加的危险因素[11]。本研究也证实移植前SF>1 000 µg/L的患儿Ⅲ～Ⅳ度aGVHD发生率显著增加。铁过载除诱导重要脏器功能障碍外，也可促进细菌、真菌等病原体生长[3]。Dadwal等[12]对447例接受allo-HSCT患者研究发现，移植前铁过载患者在移植后30 d内侵袭性真菌感染率显著升高。因此，铁过载可能不仅导致患儿皮肤、肠道、肝脏等靶器官损伤，同时激活患儿机体炎症应答，促进aGVHD的发生和发展。有研究显示铁蛋白与aGVHD发生率无明显相关[13]，甚或呈负相关[14]，这可能与预处理方案、移植物类型等有关。

HC是移植后常见并发症之一。预处理药物、GVHD、病毒感染等因素均可导致allo-HSCT患者并发HC风险增加[15]–[16]。我们发现铁过载组患儿移植后HC发生率显著高于非铁过载组，其原因可能为铁过载组aGVHD的发生率更高。患儿发生aGVHD时通常使用高强度免疫抑制剂，使病毒更易感染膀胱上皮细胞[15]。

既往研究认为接受HSCT的AML/MDS患者移植前铁过载可显著降低OS率，增加非复发病死率[2]–[4]。本研究发现移植前SF水平与SAA患儿OS率及FFS率无明显相关。Franke等[4]也认为SF水平对不同疾病移植结局的影响并不一致。与AML/MDS等恶性血液病不同，SAA属于骨髓衰竭性疾病，无病态造血和发育异常，其移植预处理方案为非清髓，而恶性血液病的一般为清髓方案。因此，恶性血液病患者在移植前接受高剂量多次化疗导致广泛器官损伤。其次，成人患者较之儿童更易发生铁过载[17]。这可能与儿童处于生长发育期，机体各组织器官对铁需求量更大有关。患者获得明确诊断到接受移植的间隔时间也是引起移植预后不良的因素之一[18]。与施圆圆等[19]研究相比，本研究中患儿确诊至移植的间隔时间更短，仅3（2，7）个月，提示对于符合allo-HSCT的SAA患儿应尽早启动移植程序，缩短输血依赖期，减低患儿铁负荷程度，减少铁过载对移植疗效的影响。

综上所述，SAA患儿移植前铁过载可显著延迟allo-HSCT后Treg细胞重建，增加HC和Ⅲ～Ⅳ度aGVHD发生率。SAA诊断明确后应缩短allo-HSCT前输血依赖期，控制铁过载，降低移植并发症的发生率，以期进一步改善SAA患儿接受移植后的长期生存质量。但本研究观察病例数少，可能存在偏倚；aGVHD和HC的发生率也与其他因素有关。后续需要更大样本量、前瞻性的研究来明确铁过载对移植疗效的影响。
